# Differentiation in the Underground Bud-Bank Structure of *Poa pratensis* Across Contrasting Alpine Grassland Habitats on the Eastern Qinghai–Tibetan Plateau

**DOI:** 10.3390/plants15152383

**Published:** 2026-08-03

**Authors:** Xiaoli Wang, Qian Wang, Hang Xu, Jiyun Li, Lili He, Zhenggang Guo, Xiaoxing Wei

**Affiliations:** 1 Qinghai Academy of Animal Science and Veterinary Medicine, Qinghai University, Xining 810016, China; 2 Laboratory for Germplasm Research and Utilization on the Qinghai–Tibetan Plateau, Qinghai University, Xining 810016, China; 3 State Key Laboratory of Sanjiangyuan Ecology and Plateau Agriculture and Animal Husbandry (Co-Founded by the Ministry and Province), Qinghai University, Xining 810016, China; 4 College of Pastoral Agriculture Science and Technology, Lanzhou University, State Key Laboratory of Herbage Improvement and Grassland Agro-Ecosystems, Engineering Research Center of Grassland Industry (Ministry of Education), Lanzhou 730000, China

**Keywords:** alpine steppe, alpine meadow, *Poa pratensis*, bud-bank structure, rhizome bud, tiller bud

## Abstract

Underground bud banks play a critical role in vegetative regeneration of alpine grassland plants, but how species-level underground regenerative structures vary across contrasting habitats remains unclear. This study investigated the widely distributed clonal grass *Poa pratensis* across alpine steppe and alpine meadow habitats on the Qinghai–Tibetan Plateau, focusing on the differentiation of underground bud density, morphological traits, ultrastructure, and associated soil environmental conditions. Compared with alpine steppe, alpine meadow populations exhibited significantly higher tiller bud density, bud length, and bud diameter, whereas rhizome buds were observed exclusively in alpine meadow. Furthermore, underground buds in alpine meadow showed larger dimensions and more developed surface appendages. Principal component and correlation analyses revealed that underground bud traits were associated with soil resource conditions, with higher soil moisture and nutrient availability corresponding to enhanced bud development. Collectively, the integrated differentiation of underground bud “quantity–type–structure” in *P. pratensis* suggests that environmental heterogeneity may shape belowground regenerative strategies of alpine grassland plants.

## 1. Introduction

Alpine grasslands on the Qinghai–Tibetan Plateau account for approximately 60–70% of the total regional area and represent one of the dominant vegetation types in this region [1]. These ecosystems play critical roles in maintaining ecological security, regulating regional hydrological processes, and supporting pastoral production [2,3,4]. However, under the combined influences of climate warming, grazing pressure, and land-use change, alpine grassland degradation has intensified continuously, and the decline in vegetation regeneration capacity has become a key constraint on ecosystem restoration [3,5].

Underground bud banks serve as an important foundation for vegetative regeneration in plants. They not only generate new shoots and rhizomes but also promote plant expansion through clonal growth, thereby playing essential roles in population persistence, post-disturbance recovery, and the maintenance of grassland stability [6,7]. Previous studies have shown that the size and structure of underground bud banks are jointly influenced by climatic conditions, soil resources, and environmental disturbances [8,9,10]. Across the Qinghai–Tibetan Plateau, substantial differences in water availability, temperature, and soil properties generate pronounced environmental heterogeneity [11,12]. Such heterogeneity may alter plant vegetative regeneration strategies and further lead to habitat-dependent variation in underground bud-bank characteristics [10,13]. Alpine steppe and alpine meadow represent two typical alpine grassland types on the Qinghai–Tibetan Plateau. Alpine meadows generally exhibit higher soil moisture and nutrient availability, whereas alpine steppes are characterized by stronger water limitation and environmental stress [1,12]. This natural ecological gradient from alpine steppe to alpine meadow therefore provides an ideal framework for investigating habitat differentiation in underground regeneration structures.

In clonal grasses, different underground bud types are typically associated with distinct ecological functions. Tiller buds are mainly related to local persistence and compact growth, whereas rhizome buds facilitate lateral expansion and spatial occupation [8,14,15]. Therefore, variation in underground bud composition and structural characteristics may reflect shifts in belowground regeneration strategies under different habitat conditions [8]. Although previous studies have examined changes in underground bud bank density and abundance [8,15], these studies have largely focused on community-level patterns, whereas species-level variation in underground bud type composition, morphology, and ultrastructure across contrasting environments remains poorly understood. Community-scale analyses are often strongly influenced by differences in species composition, making it difficult to reveal the true variation in underground regeneration structures of individual dominant species across contrasting environments. Moreover, existing studies on underground bud morphology and regeneration structures have primarily focused on temperate grasslands and wetland ecosystems [16,17,18]. Direct evidence remains limited regarding whether contrasting alpine habitats are associated with differentiation in underground bud-bank structure and belowground regeneration patterns of clonal grass species.

*Poa pratensis* is a widespread perennial clonal grass species in alpine grasslands on the Qinghai–Tibetan Plateau and is widely used in artificial grassland establishment and ecological restoration [19,20]. The species possesses strong clonal growth ability, with tiller buds mainly contributing to local persistence and rhizomes facilitating spatial expansion, thereby forming abundant underground bud banks and enabling efficient vegetative regeneration [21,22]. Because *P. pratensis* is widely distributed in both alpine steppe and alpine meadow habitats, it provides an ideal model for examining habitat-related variation in underground bud composition and belowground regeneration structure. Therefore, along the natural ecological gradient from alpine steppe to alpine meadow in the eastern Qinghai–Tibetan Plateau, this study investigated habitat-associated variation in underground bud-bank abundance, composition, morphology, and ultrastructure of *P. pratensis* and examined their relationships with associated soil environmental conditions. We proposed the following hypotheses: (1) *P. pratensis* would exhibit greater underground bud abundance and larger bud size in alpine meadow than in alpine steppe, and (2) contrasting alpine habitats would be associated with differentiation in underground bud type composition, morphology, and belowground regeneration structures of *P. pratensis*.

## 2. Materials and Methods

### 2.1. Study Site Description

The study sites were located in the eastern and northern parts of Qinghai Province, China (Figure 1). To minimize the potential confounding effects between grassland type and geographic region, both alpine steppe and alpine meadow sites were established in eastern and northern Qinghai. The elevation of the study sites ranged from 3033 to 3616 m a.s.l., forming a natural ecological gradient from alpine steppe to alpine meadow. Due to differences in elevation and regional climatic conditions, the study area exhibited pronounced environmental heterogeneity. According to regional meteorological records, mean annual temperature (MAT) ranged from 0.6 to 2.9 °C, whereas mean annual precipitation (MAP) ranged from 320 to 480 mm. Overall, alpine meadow sites were characterized by relatively higher precipitation and more humid climatic conditions, whereas alpine steppe sites were comparatively drier. Grassland types were classified according to community species composition, vegetation structural characteristics, and the regional alpine grassland classification system [23,24]. Alpine steppe sites were mainly dominated by Poaceae species, whereas alpine meadow sites were dominated by Cyperaceae and Poaceae species. *P. pratensis* was distributed across all study sites and was selected as the target species in this study. All study sites had been fenced for approximately three years before sampling to exclude livestock grazing. During this period, no intensive anthropogenic disturbances, such as mowing, trampling, or vegetation restoration activities, were conducted within the sampling areas. The selected sites represented relatively stable alpine grassland communities under natural environmental conditions. Detailed geographical coordinates, elevation, and climatic characteristics of the six study sites are provided in Supplementary Table S1. The study area map (Figure 1) was created using ArcGIS 10.8 (Esri, Redlands, CA, USA).

### 2.2. Bud Sampling and Processing

Underground buds of *P. pratensis* were sampled using a quadrat excavation method during the early green-up stage (10 May 2023), when overwintering buds had not yet extensively sprouted [6]. This timing allowed assessment of the potential vegetative regeneration capacity while minimizing phenological differences among habitats. For each grassland type, three independent sites were established. Within each site, three relatively homogeneous vegetation patches were selected at intervals of approximately 50 m. In each patch, three 0.25 m × 0.25 m quadrats were randomly established, with adjacent quadrats separated by approximately 10 m. All quadrats were located away from roads, bare ground, rodent burrows, trampling areas, and habitat edges to minimize the influence of local disturbances on sampling results. Within each quadrat, aboveground vegetation was clipped at the soil surface, and surface litter was removed. The soil was then excavated vertically along the quadrat boundaries to a depth of 0–20 cm, and the entire soil block containing roots, rhizomes, and underground buds was collected as a bud bank sample. The 0–20 cm layer was selected because it represents the primary distribution zone of underground buds and vegetative reproductive organs in alpine grasslands [25,26]. Care was taken to maintain the integrity of rhizomes and buds during excavation to prevent mechanical damage. Excavated samples were placed in labeled mesh bags and transported to the laboratory, where they were gently washed to remove adhering soil. Underground buds were manually separated and classified into tiller buds and rhizome buds based on their attachment position and morphological characteristics. The number of buds in each quadrat was recorded and converted to bud density (buds m^−2^). For morphological measurements, intact buds were randomly selected from each quadrat, and bud length and diameter were measured using a digital caliper.

### 2.3. Microscopic Observation of Bud Morphology and Ultrastructure

Underground buds used for morphological and ultrastructural observations were immediately fixed after collection in FAA (formalin–acetic acid–alcohol) solution (70% ethanol–glacial acetic acid–formaldehyde = 18:1:1, *v*/*v*/*v*) and stored at 4 °C. Overall bud morphology was examined using a stereomicroscope (Nikon SMZ18, Nikon Corporation, Tokyo, Japan) at approximately 10× magnification, and images were captured and analyzed using NIS-Elements software (Version 4.40.00; Nikon Corporation, Tokyo, Japan). The surface ultrastructure of underground buds was examined using scanning electron microscopy (SEM). Before SEM observation, fixed bud samples were naturally air-dried. Representative young leaves, scale leaves, and their surface appendages from underground buds were carefully selected for ultrastructural examination. SEM observations were performed using JSM-7900F and JSM-6610LV microscopes (JEOL Ltd., Tokyo, Japan) at approximately 350× magnification. Particular attention was given to differences in the microstructure of underground buds among habitats, including epidermal morphology of young leaves, scale leaf morphology, and surface appendages.

### 2.4. Soil Sampling and Soil Property Measurements

To ensure spatial correspondence between soil properties and underground bud bank characteristics, soil samples were collected simultaneously with underground bud sampling adjacent to each bud sampling quadrat. Soil was sampled from the 0–20 cm layer using a 5 cm diameter soil auger. Five sampling points were arranged around each quadrat in an “S”-shaped pattern and combined into one composite sample. Each composite soil sample corresponded to the same quadrat used for underground bud-bank sampling. The 0–20 cm layer was selected because it represents the primary distribution zone of underground buds, rhizomes, fine roots, and other vegetative reproductive organs in alpine grasslands and corresponds to the biologically active soil layer with high moisture, nutrient availability, and microbial activity [8,25]. Fresh soil samples were passed through a 2 mm sieve to remove plant residues, roots, and gravel. A portion of each soil sample was oven-dried at 105 °C to constant weight to determine soil water content (SWC) using the gravimetric method. Soil bulk density (BD) was measured using the core method [27]. Soil pH was determined potentiometrically in a 1:2.5 soil-to-water suspension. Soil organic carbon (SOC) was measured using the dichromate oxidation method [28]. Available phosphorus (AP) was determined using the molybdenum blue colorimetric method following NaHCO_3_ extraction [28]. Ammonium nitrogen (NH_4_^+^–N) and nitrate nitrogen (NO_3_^−^–N) were extracted with 2 mol L^−1^ KCl and quantified using a continuous flow analyzer [29].

### 2.5. Statistical Analysis

All statistical analyses were performed in R version 4.3.1(R Core Team, Vienna, Austria) [30]. Data normality was assessed using the Shapiro–Wilk test, and homogeneity of variance was evaluated using Levene’s test. Variables that did not meet the assumptions of normality or homoscedasticity were log-transformed when necessary. To evaluate the effects of grassland type on underground bud traits and soil physicochemical properties of *P. pratensis*, linear mixed-effects models were applied. Grassland type was treated as a fixed effect, whereas site was included as a random effect to account for the hierarchical structure of the sampling design and spatial dependence among observations within the same site. The model structure was as follows:

Trait or soil variable ~ Grassland type + (1 | Site)

Patches and quadrats were treated as nested spatial subsampling units used to characterize within-site spatial variability rather than independent biological replicates. Therefore, patch effects were not included as an additional random effect in the mixed-effects models. Statistical significance was determined at *p* < 0.05, and all data are presented as mean ± SE. Points in the figures represent quadrat-level observations, whereas statistical inference was based on the results of linear mixed-effects models with site included as a random effect.

Principal component analysis (PCA) was performed using the *stats* package to comprehensively characterize differences in soil environmental conditions between grassland types. All variables were standardized prior to analysis to eliminate the influence of differences in measurement units. Relationships between underground bud traits and soil factors were evaluated using Spearman correlation analysis and visualized using the *corrplot* package (version 0.92). These correlation analyses were used primarily as exploratory analyses to examine associations between underground bud traits and soil variables rather than to infer causality. Because rhizome buds were observed exclusively in alpine meadow and were completely absent in alpine steppe, correlation analyses were conducted only for tiller bud traits. All figures were generated using the R package *ggplot2* (version 3.5.1).

## 3. Results

### 3.1. Effects of Grassland Type on Bud Traits of Poa pratensis

Alpine meadow exhibited significantly greater bud density, length, and diameter than alpine steppe (Figure 2). Specifically, tiller bud density in alpine meadow was 65.1% higher than that in alpine steppe (Figure 2A). Tiller bud length increased by 75.1% in alpine meadow compared with alpine steppe (Figure 2B), whereas tiller bud diameter increased by 57.1% (Figure 2C). Rhizome buds were observed exclusively in alpine meadow sites, whereas no rhizome buds were detected in alpine steppe sites (Table 1). In alpine meadow, rhizome bud density, length, and diameter were 186.07 ± 2.44 buds m^−2^, 21.18 ± 0.26 mm, and 1.17 ± 0.01 mm, respectively.

### 3.2. Effects of Grassland Type on the Bud Morphological Structure of Poa pratensis

Under stereomicroscopic observation, distinct differences in the morphological structure of underground buds of *P. pratensis* were observed between alpine steppe and alpine meadow (Figure 3). Tiller buds from both grassland types were primarily composed of young leaves enclosed by scale leaves (Figure 3A,B). Rhizome buds were observed exclusively in alpine meadow and were characterized by elongated rhizome nodes and relatively well-developed bud structures (Figure 3C). Scanning electron microscopy further revealed clear differences in the surface ultrastructure of underground buds between grassland types (Figure 4). The surface of young leaves in alpine steppe appeared relatively smooth (Figure 4A), whereas distinct trichome structures were observed on the surfaces of young leaves in both tiller buds and rhizome buds from alpine meadow sites (Figure 4B,C).

### 3.3. Effects of Grassland Type on Soil Physicochemical Properties

Significant differences in soil physicochemical properties were observed between alpine steppe and alpine meadow (Figure 5). Compared with alpine steppe, SWC in alpine meadow increased by 40.3% (*p* < 0.001; Figure 5A), whereas BD decreased by 11.2% (*p* < 0.001; Figure 5B). In addition, soil pH in alpine meadow was significantly lower than that in alpine steppe (*p* < 0.01; Figure 5C). Regarding soil nutrient properties, SOC, NH_4_^+^–N, and AP in alpine meadow were 58.1%, 17.0%, and 29.5% higher, respectively, than those in alpine steppe (*p* < 0.001; Figure 5D,E,G). In contrast, NO_3_^−^–N content was significantly higher in alpine steppe than in alpine meadow (*p* < 0.001; Figure 5F).

### 3.4. Principal Component Analysis of Soil Environments Across Grassland Types

PCA revealed a clear separation in soil environmental characteristics between alpine steppe and alpine meadow (Figure 6). The first two principal components explained 82.8% of the total variation, with PC1 and PC2 accounting for 65.3% and 17.5% of the variation, respectively. Along the PC1 axis, sample points from alpine steppe were mainly distributed on the negative side of the axis and were associated with higher BD, pH, and NO_3_^−^–N. In contrast, sample points from alpine meadow were mainly distributed on the positive side of PC1 and were associated with higher SWC, SOC, AP, and NH_4_^+^–N.

### 3.5. Relationships Between Underground Bud Traits and Soil Factors

Rhizome buds were observed only in alpine meadow; thus, correlation analyses were performed exclusively for tiller bud traits. Spearman correlation analysis revealed significant relationships between tiller bud traits and soil environmental factors (Figure 7). Tiller bud density, length, and diameter were significantly positively correlated with SWC, SOC, NH_4_^+^–N, and AP (*p* < 0.001), whereas they were significantly negatively correlated with BD and NO_3_^−^–N (*p* < 0.001). In addition, soil pH also showed significant negative correlations with tiller bud traits (*p* < 0.05 or *p* < 0.01). To further quantify the relationships between tiller bud density and key soil resource variables, scatter plot analyses were conducted across the two grassland types. Tiller bud density showed significant positive relationships with SWC, SOC, NH_4_^+^–N, and AP, with Spearman correlation coefficients ranging from 0.65 to 0.77 (*p* < 0.001; Figure S1). Among these variables, SOC, AP, and SWC exhibited relatively stronger associations with tiller bud density, whereas NH_4_^+^–N showed a comparatively weaker association.

## 4. Discussion

Previous studies on underground bud banks have mainly focused on the community scale [8], primarily emphasizing variations in bud number and density [26]. In contrast, the present study focused on *P. pratensis*, a dominant species in alpine grasslands, and further revealed systematic differences in underground bud type composition, bud morphology, and ultrastructure across contrasting alpine habitats.

Our results demonstrated significant differences in the underground bud bank of *P. pratensis* between alpine steppe and alpine meadow. Compared with alpine steppe, *P. pratensis* in alpine meadow exhibited significantly greater tiller bud density, bud length, and bud diameter (Figure 2), indicating a stronger belowground regeneration capacity in alpine meadow and thereby supporting our first hypothesis. Previous studies have shown that resource-rich environments generally favor the formation of larger underground bud banks in clonal plants, thereby enhancing plant regeneration and persistence [31,32,33], and our findings are broadly consistent with this pattern. In the present study, alpine meadow was primarily associated with higher soil moisture, soil organic carbon, and available phosphorus, whereas alpine steppe was more closely associated with higher soil bulk density and pH (Figure 6), indicating contrasting resource environments between the two alpine habitats. Furthermore, higher soil moisture and nutrient availability in alpine meadow were positively correlated with underground bud density, bud length, and bud diameter (Figure 7), which may help explain its higher belowground regenerative potential [18,24]. In contrast, the relatively lower resource availability in alpine steppe may constrain underground bud development, which was consistent with its lower belowground regenerative potential [27,28]. However, because multiple soil variables varied simultaneously between habitats and were strongly intercorrelated, this study could not distinguish the independent effects of individual soil factors. Therefore, the observed relationships are more likely to reflect the combined effects of integrated habitat resource conditions rather than the independent influence of any single soil variable.

Beyond the increase in underground bud abundance, rhizome buds were observed exclusively in alpine meadow and were not detected in alpine steppe sites. This “presence–absence” pattern further reflects a clear differentiation in underground bud type composition between contrasting alpine habitats [14,34]. These findings suggest that alpine environments may influence not only underground bud abundance but also the composition of belowground regeneration types and underground expansion patterns, thereby supporting our second hypothesis. Previous studies have shown that rhizomatous clonal organs are often associated with enhanced lateral expansion and increased resource foraging capacity and are more likely to develop and persist under relatively favorable resource conditions [31,35]. Therefore, the exclusive occurrence of rhizome buds in alpine meadow may indicate that *P. pratensis* exhibits different belowground regeneration patterns across alpine habitats: alpine meadow may favor expansive clonal growth, whereas alpine steppe may exhibit relatively conservative local renewal characteristics [31,34,36,37]. However, the formation and detection of rhizome buds may also be influenced by other factors, including plant developmental stage [34], seasonal variation [38], grazing history [39], and disturbance intensity [10]. Future studies integrating multi-season observations and controlled experiments are needed to further clarify the mechanisms underlying habitat-related differences in rhizome bud formation.

Differences in underground bud morphology and ultrastructure further supported this habitat differentiation pattern. In the present study, underground buds in alpine meadow exhibited larger bud length and diameter and showed more pronounced surface appendages under scanning electron microscopy observations (Figure 3 and Figure 4). These characteristics may be associated with greater resource storage potential and stress tolerance of underground buds in alpine meadow. Alpine grassland ecosystems are generally characterized by strong low-temperature stress, frequent freeze–thaw events, and pronounced seasonal fluctuations in soil moisture [35,40], meaning that underground buds must withstand substantial environmental stress during overwintering and spring regrowth. Under such conditions, larger buds may possess greater storage reserves and higher germination potential, which may contribute to early-season shoot emergence and regeneration potential [41,42]. Previous studies have reported that certain protective surface structures in alpine or cold-environment plants may help maintain local microenvironmental stability and alleviate damage caused by low temperature or desiccation to young tissues [43,44]. Furthermore, given the known genetic variation [45], polyploidy [46], and environmental responsiveness of *Poa pratensis* [45], the observed differences in underground bud morphology and ultrastructure may result from environmental responses, genetic divergence, or their combined effects. Therefore, the relative contributions of environmental plasticity and genetic variation, as well as the specific physiological functions of these structural traits, require further investigation. Moreover, considering the limited number of sampling sites in this study and the high environmental heterogeneity of the Qinghai–Tibetan Plateau, future studies with broader spatial coverage and more extensive sampling are needed to further evaluate the generality and applicability of underground bud-bank differentiation patterns across alpine habitats.

## 5. Conclusions

This study revealed habitat differentiation in the underground regeneration structure of alpine clonal plants from three aspects, including underground bud quantity, type composition, and ultrastructural characteristics. The results showed that, compared with alpine steppe, *P. pratensis* in alpine meadow exhibited higher tiller bud density, larger bud size, and formed rhizome buds and other expansive underground regeneration structures, whereas rhizome buds were absent in alpine steppe. In addition, underground buds in alpine meadow showed more pronounced surface appendages and were closely associated with higher soil moisture and nutrient conditions. These results indicated that significant differences existed in underground bud quantity, type composition, and structural characteristics of *P. pratensis* among different alpine habitats. Alpine meadow was associated with the formation of more expansive clonal structures, whereas alpine steppe tended to maintain a more conservative local renewal pattern. Such differentiation in underground regeneration structure may reflect the long-term adaptation of *P. pratensis* to contrasting resource conditions and environmental stresses across alpine habitats. These findings suggest that habitat conditions should be fully considered when investigating vegetative regeneration processes and underground regeneration structures in alpine grasslands.

## Figures and Tables

**Figure 1:**
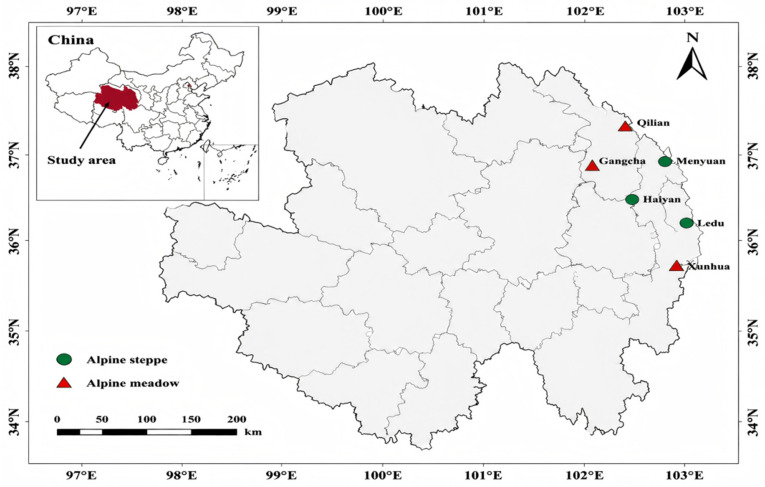
Distribution of the study sites. Green circles and red triangles indicate alpine steppe and alpine meadow sites, respectively.

**Figure 2:**
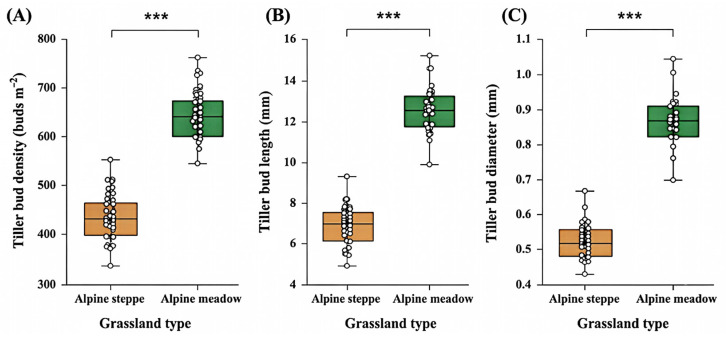
Effects of grassland type on the tiller bud traits of *Poa pratensis*: (**A**) tiller bud density; (**B**) tiller bud length; (**C**) tiller bud diameter. Boxes represent the interquartile range, horizontal lines within boxes indicate the median, whiskers indicate the range excluding outliers, and points represent individual observations. Asterisks (***) indicate significant differences between alpine steppe and alpine meadow at *p* < 0.001.

**Figure 3:**
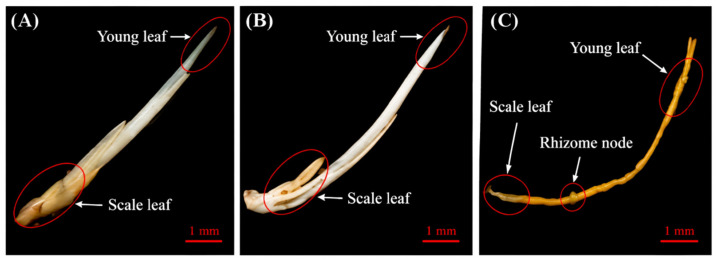
Morphological characteristics of underground buds of *Poa pratensis* in alpine grasslands under a stereomicroscope: (**A**) tiller bud in alpine steppe; (**B**) tiller bud in alpine meadow; (**C**) rhizome bud in alpine meadow. Arrows indicate young leaves, scale leaves, and rhizome nodes. Scale bar = 1 mm.

**Figure 4:**
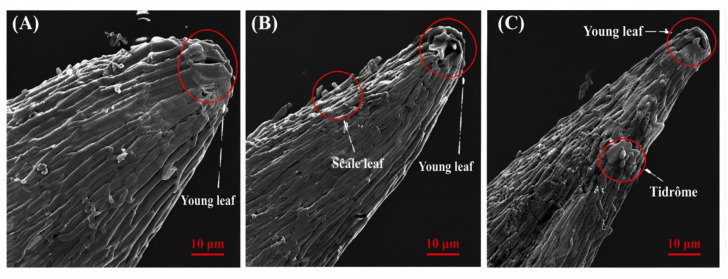
Scanning electron micrographs showing the surface ultrastructure of underground buds of *Poa pratensis* in alpine grasslands: (**A**) tiller bud in alpine steppe; (**B**) tiller bud in alpine meadow; (**C**) rhizome bud in alpine meadow. Arrows indicate trichomes and young leaf structures. Scale bar = 10 μm.

**Figure 5:**
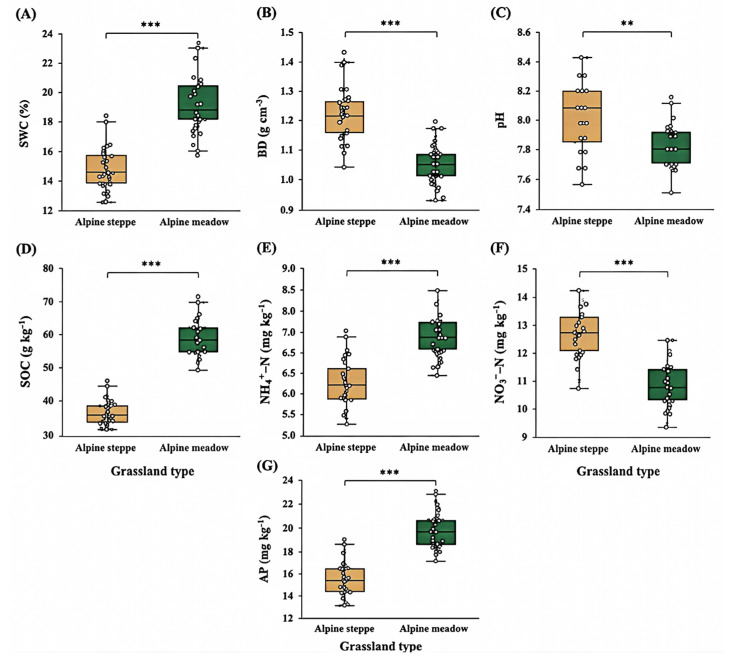
Effects of grassland type on soil physicochemical properties associated with *Poa pratensis* across alpine grasslands: (**A**) SWC, soil water content; (**B**) BD, soil bulk density; (**C**) pH; (**D**) SOC, soil organic carbon; (**E**) NH_4_^+^–N, ammonium nitrogen; (**F**) NO_3_^−^–N, nitrate nitrogen; and (**G**) AP, available phosphorus. Boxes represent the interquartile range, horizontal lines within boxes indicate the median, whiskers indicate the range excluding outliers, and points represent individual observations. Asterisks indicate significant differences between alpine steppe and alpine meadow (** *p* < 0.01; *** *p* < 0.001).

**Figure 6:**
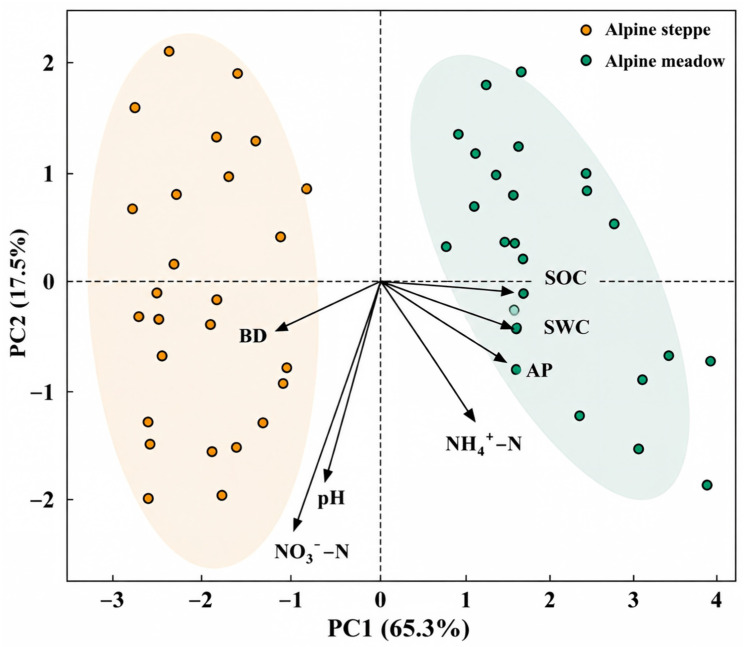
Principal component analysis (PCA) of soil environmental variables associated with *Poa pratensis* across alpine steppe and alpine meadow habitats. Arrows indicate the direction and strength of correlations between soil variables and principal components. SOC, soil organic carbon; SWC, soil water content; AP, available phosphorus; NH_4_^+^–N, ammonium nitrogen; NO_3_^−^–N, nitrate nitrogen; BD, soil bulk density. Ellipses represent 95% confidence intervals for each habitat type.

**Figure 7:**
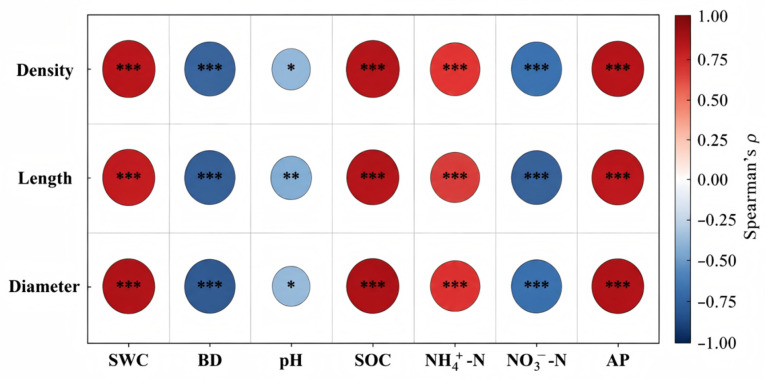
Spearman correlation analysis between tiller bud traits of *Poa pratensis* and soil physicochemical properties in alpine grasslands. Circle color indicates the direction of the correlation (red: positive; blue: negative), and circle size represents the strength of the correlation coefficient. Asterisks indicate significant correlations (* *p* < 0.05, ** *p* < 0.01, *** *p* < 0.001). SWC, soil water content; BD, soil bulk density; SOC, soil organic carbon; NH_4_^+^–N, ammonium nitrogen; NO_3_^−^–N, nitrate nitrogen; AP, available phosphorus.

**Table 1:** Rhizome bud traits of *Poa pratensis* in alpine grasslands.

Grassland Type	Rhizome Bud Density (Buds m^−2^)	Rhizome Bud Length (mm)	Rhizome Bud Diameter (mm)
Alpine steppe	0	—	—
Alpine meadow	186.07 ± 2.44	21.18 ± 0.26	1.17 ± 0.01

Note: Rhizome buds were not detected in alpine steppe sites. Values are presented as mean ± SE.

## Data Availability

The datasets generated and/or analyzed during the current study are available from the corresponding author upon reasonable request.

## Supplementary Materials

The following supporting information can be downloaded at: https://www.mdpi.com/article/10.3390/plants15152383/s1, Figure S1: Relationships between tiller bud density of *Poa pratensis* and soil environmental variables across alpine steppe and alpine meadow habitats. (a) Soil water content (SWC); (b) soil organic carbon (SOC); (c) ammonium nitrogen (NH_4_^+^–N); and (d) available phosphorus (AP). Each point represents an individual quadrat observation (n = 54). Triangles indicate alpine steppe, and circles indicate alpine meadow. Gray lines represent overall linear trends based on all quadrat observations across the two grassland types. Spearman correlation coefficients were calculated using all quadrat observations, and all correlations shown were significant (*p* < 0.001); Table S1: Geographical and climatic characteristics of the six study sites.

## References

[1] Li M., Zhang X., Wu J., Li W., Zhao Y., Zhou H., Li X. (2020). Assessment of the vulnerability of alpine grasslands on the Qinghai–Tibetan Plateau. PeerJ.

[2] Yao T.D., Thompson L.G., Mosbrugger V., Zhang F., Ma Y.M., Luo T.X., Xu B.Q., Yang X.X., Joswiak D.R., Wang W.C. (2012). Third pole environment (TPE). Environ. Dev..

[3] Dong S.K., Shang Z.H., Gao J.X., Boone R.B. (2020). Enhancing sustainability of grassland ecosystems through ecological restoration and grazing management in an era of climate change on Qinghai-Tibetan Plateau. Agric. Ecosyst. Environ..

[4] Zhou H., Yang X., Zhou C., Shao X., Shi Z., Li H., Su H., Qin R., Chang T., Hu X. (2023). Alpine grassland degradation and its restoration in the Qinghai–Tibet Plateau. Grasses.

[5] Zhu Q., Chen H., Peng C., Liu J.X., Piao S.L., He J.S., Wang S.P., Zhao X.Q., Zhang J., Fang X.Q. (2023). An early warning signal for grassland degradation on the Qinghai-Tibetan Plateau. Nat. Commun..

[6] Klimeš J. (2007). Bud banks and their role in vegetative regeneration—A literature review and proposal for simple classification and assessment. Perspect. Plant Ecol. Evol. Syst..

[7] Ott J.P., Hartnett D.C. (2012). Contrasting bud bank dynamics of two co-occurring grasses in tallgrass prairie: Implications for grassland dynamics. Plant Ecol..

[8] Ott J.P., Klimešová J., Hartnett D.C. (2019). The ecology and significance of below-ground bud banks in plants. Ann. Bot..

[9] Wu J., Wang Y.C., Ma Q., Liu Z.M. (2020). Roles of aboveground vegetation, soil properties, and disturbance in determining belowground bud bank in sand dune ecosystems. Environ. Exp. Bot..

[10] Li W.C., Huang A.P., Zhou T.C., Liu M., Ma S.J., Zhao N.N., Wang X.T., Sun J. (2023). Patterns and drivers of the belowground bud bank in alpine grasslands on the Qinghai-Tibet Plateau. Front. Plant Sci..

[11] Yang Y.H., Fang J.Y., Pan Y.D., Ji C.J. (2009). Aboveground biomass in Tibetan grasslands. J. Arid Environ..

[12] Wang Y.F., Lv W.W., Xue K., Wang S.P., Zhang L.R., Hu R.H., Zeng H., Xu X.L., Li Y.M., Jiang L.L. (2022). Grassland changes and adaptive management on the Qinghai–Tibetan Plateau. Nat. Rev. Earth Environ..

[13] Wu J., Hou X.Z., Xu L., Zhou Q.L., Wang Y.C., Guo Z.W., Adomako M.O., Ma Q. (2024). Belowground bud banks and land use change: Roles of vegetation and soil properties in mediating the composition of bud banks in different ecosystems. Front. Plant Sci..

[14] Klimeš L., de Kroon H., van Groenendael J. (1997). Clonal plant architecture: A comparative analysis of form and function. The Ecology and Evolution of Clonal Plants.

[15] Benson E.J., Hartnett D.C., Mann K.H. (2004). Belowground bud banks and meristem limitation in tallgrass prairie plant populations. Am. J. Bot..

[16] Klimešová J., Martínková J., Ottaviani G. (2018). Belowground plant functional ecology: Towards an integrated perspective. Funct. Ecol..

[17] Yu F.H., Wang N., He W.M., Chu Y., Dong M. (2008). Adaptation of rhizome connections in drylands: Increasing tolerance of clones to wind erosion. Ann. Bot..

[18] Fang T., Ye D., Gao J.J., Luo F.L., Zhu Y.J., Yu F.H. (2024). Responses of belowground bud bank density of geophytes to environmental perturbations: A meta-analysis. J. Plant Ecol..

[19] Ma Y.L., Liu Y.F., Rodrigo-Comino J., López-Vicente M., Wu G.L. (2024). Mixed-cultivation grasslands enhance runoff generation and reduce soil loss in the restoration of degraded alpine hillsides. Hydrol. Earth Syst. Sci..

[20] Xing Y.F., Shi J.J., Ma Y., Ou W.Y., Liu Q.Q., Lyu L.Y., Zhang H.R., Cai Z.C. (2024). Keystone species and driving factors of artificial grassland on the Qinghai–Tibetan Plateau, China. Diversity.

[21] Moser L.E., Anderson S.R., Miller R.W. (1968). Rhizome and tiller development of Kentucky bluegrass (*Poa pratensis* L.) as influenced by photoperiod, cold treatment, and variety. Agron. J..

[22] Ran F., Yuan Y.J., Bai X.M., Li C.N., Li J.X., Chen H. (2023). Carbon and nitrogen metabolism affects Kentucky bluegrass rhizome expansion. BMC Plant Biol..

[23] Ren J.Z., Hu Z.Z., Zhao J., Zhang D.G., Hou F.J., Lin H.L., Mu X.D. (2008). A grassland classification system and its application in China. Rangel. J..

[24] He P.F., Shi Y.L., Ding H.Y., Yang F.W. (2023). Classification and transition of grassland in Qinghai, China, from 1986 to 2020 with Landsat archives on Google Earth Engine. Land.

[25] Ding X.J., Su P.X., Zhou Z.J., Shi R. (2019). Belowground bud bank distribution and aboveground community characteristics along different moisture gradients of alpine meadow in the Zoige Plateau, China. Sustainability.

[26] Dalgleish H.J., Hartnett D.C. (2006). Below-ground bud banks increase along a precipitation gradient of the North American Great Plains: A test of the meristem limitation hypothesis. New Phytol..

[27] Blake G.R., Hartge K.H., Klute A. (1986). Bulk density. Methods of Soil Analysis: Part 1. Physical and Mineralogical Methods.

[28] Bao S.D. (2000). Soil and Agricultural Chemistry Analysis.

[29] Keeney D.R., Nelson D.W., Page A.L., Miller R.H., Keeney D.R. (1982). Nitrogen—Inorganic forms. Methods of Soil Analysis: Part 2. Chemical and Microbiological Properties.

[30] R Core Team (2023). R: A Language and Environment for Statistical Computing.

[31] de Kroon H., Hutchings M.J. (1995). Morphological plasticity in clonal plants: The foraging concept reconsidered. J. Ecol..

[32] Liu F.H., Liu J., Dong M. (2016). Ecological consequences of clonal integration in plants. Front. Plant Sci..

[33] Herben T., Klimešová J., Chytrý M. (2018). Effects of disturbance frequency and severity on plant traits: An assessment across a temperate flora. Funct. Ecol..

[34] Klimešová J., Martínková J., Pausas J.G., de Moraes M.G., Herben T., Yu F.H., Puntieri J., Vesk P.A., de Bello F., Janeček Š. (2019). Handbook of standardized protocols for collecting plant modularity traits. Perspect. Plant Ecol. Evol. Syst..

[35] Wang J.Y., Xu T.T., Wang Y., Li G.Y., Abdullah I., Zhong Z.W., Liu J.S., Zhu W.Y., Wang L., Wang D. (2021). A meta-analysis of effects of physiological integration in clonal plants under homogeneous vs. heterogeneous environments. Funct. Ecol..

[36] Ottaviani G., Martínková J., Herben T., Pausas J.G., Klimešová J. (2017). On plant modularity traits: Functions and challenges. Trends Plant Sci..

[37] Wu J., Hou X.Z., Zhu J.L., Miao R.H., Adomako M.O. (2025). Nitrogen addition and drought impose divergent effects on belowground bud banks of grassland community: A meta-analysis. Front. Plant Sci..

[38] Pu W., Yu Y., Shi X., Shao Y., Ye B., Chen Y., Song Q., Shen J., Li H. (2024). The Effect of Seasonal and Annual Variation on the Quality of *Polygonatum cyrtonema* Hua Rhizomes. Plants.

[39] Qian J., Guo Z., Muraina T.O., Te N., Griffin-Nolan R.J., Song L., Xu C., Yu Q., Zhang Z., Luo W. (2022). Legacy effects of a multi-year extreme drought on belowground bud banks in rhizomatous vs bunchgrass-dominated grasslands. Oecologia.

[40] Fu G., Shen Z.X., Zhang X.Z. (2018). Increased precipitation has stronger effects on plant production of an alpine meadow than does experimental warming in the Northern Tibetan Plateau. Agric. For. Meteorol..

[41] Vesk P.A., Westoby M. (2004). Sprouting ability across diverse disturbances and vegetation types worldwide. J. Ecol..

[42] Pausas J.G., Lamont B.B., Paula S., Appezzato-da-Glória B., Fidelis A. (2018). Unearthing belowground bud banks in fire-prone ecosystems. New Phytol..

[43] Körner C. (1999). Alpine Plant Life: Functional Plant Ecology of High Mountain Ecosystems.

[44] Neuner G. (2014). Frost resistance in alpine woody plants. Front. Plant Sci..

[45] Bushman B.S., Robbins M.D., Thorsted K., Robins J.G., Warnke S.E., Martin R., Harris-Shultz K. (2021). Transcript responses to drought in Kentucky bluegrass (*Poa pratensis* L.) germplasm varying in their tolerance to drought stress. Environ. Exp. Bot..

[46] Wei L., Zhang C., Dong Q., Yu Y., Yang X. (2020). Characterization of the complete chloroplast genome of *Poa pratensis* L. cv. Qinghai (Gramineae). Mitochondrial DNA Part B Resour..

