# Supplementary material for: Differentiation in the Underground Bud-Bank Structure of Poa pratensis Across Contrasting Alpine Grassland Habitats on the Eastern Qinghai–Tibetan Plateau
Source: Plants (Basel). 2026 Aug 3;15(15):2383. doi: 10.3390/plants15152383 (PMC13468222; doi:10.3390/plants15152383)
Supplement: Supplementary file 1 [file plants-15-02383-s001.zip › plants-4448301-supplementary.pdf]

Table S1. Geographical and climatic characteristics of the six study sites.

Site	Grassland Type	Elevation (m)	Longitude (°E)	Latitude (°N)	MAT (°C)	MAP (mm)
Ledu	Alpine steppe	3033	102°30′38″	36°29′56″	2.9	335
Menyuan	Alpine steppe	3286	101°37′50″	37°23′10″	1.2	320
Haiyan	Alpine steppe	3332	100°54′20″	36°53′11″	1.1	410
Xunhua	Alpine meadow	3267	102°29′15″	35°51′32″	2.5	420
Gangcha	Alpine meadow	3386	100°08′45″	37°19′26″	1.0	480
Qilian	Alpine meadow	3616	100°39′58″	38°02′28″	0.6	436

MAT, mean annual temperature; MAP, mean annual precipitation.

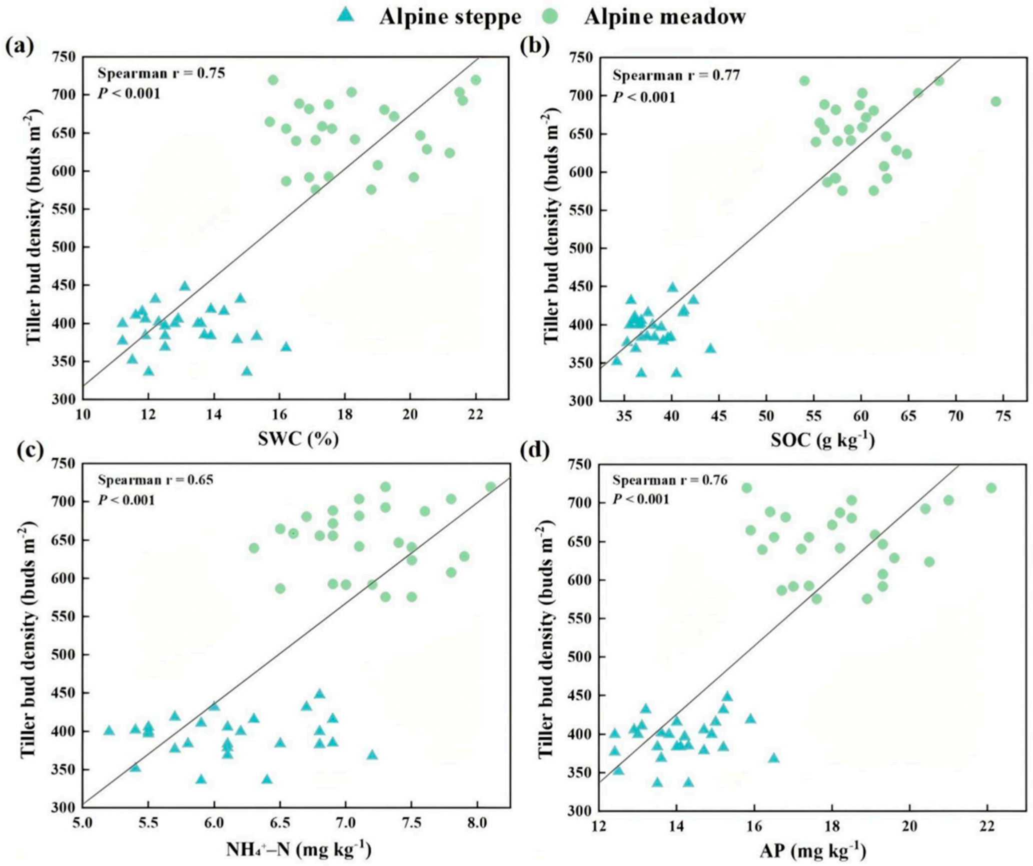

Figure S1. Relationships between tiller bud density of *Poa pratensis* and soil environmental variables across alpine steppe and alpine meadow habitats. (**a**) Soil water content (SWC); (**b**) soil organic carbon (SOC); (**c**) ammonium nitrogen (NH_4^+–N); and (**d**) available phosphorus (AP). Each point represents an individual quadrat observation (n = 54). Triangles indicate alpine steppe and circles indicate alpine meadow. Gray lines represent overall linear trends based on all quadrat observations across the two grassland types. Spearman correlation coefficients were calculated using all quadrat observations, and all correlations shown were significant ($p < 0.001$).
